# Retracted: Topical Treatment with Xiaozheng Zhitong Paste (XZP) Alleviates Bone Destruction and Bone Cancer Pain in a Rat Model of Prostate Cancer-Induced Bone Pain by Modulating the RANKL/RANK/OPG Signaling

**DOI:** 10.1155/2020/1938781

**Published:** 2020-12-18

**Authors:** Evidence-Based Complementary and Alternative Medicine

*Evidence-Based Complementary and Alternative Medicine* has retracted the article titled “Topical Treatment with Xiaozheng Zhitong Paste (XZP) Alleviates Bone Destruction and Bone Cancer Pain in a Rat Model of Prostate Cancer-Induced Bone Pain by Modulating the RANKL/RANK/OPG Signaling” [1]. As raised on PubPeer [2], the article was found to contain images with signs of duplication and manipulation in Figures 2 and 5.

(i) The medium dose panel in Figure 2(b), showing 3D micro-CT images of the tibia, shows high similarity to parts of the Placebo and High-dose panels and may be a composite image:
  1. The upper part of the Medium dose is similar to the upper part of the Placebo panel.
  2. The lower part of the Medium-dose panel is similar to the lower part of the High-dose panel.
(ii) Figures 5(a) and 5(b) include repeated images:
  1. In Figure 5(a), the bottom right of the Control panel is the same as the top left of the Medium-dose panel.
  2. In Figure 5(b), the top right of the Low-dose panel is the same as the bottom left of the High-dose panel.
(iii) Comparing the submitted figures to the published figures:
  1. In Figure 2(b), the 3D micro-CT images of the tibias are not the same between the submitted, accepted, and published versions of the article and show clear signs of editing.
  2. In Figure 5(b), the High-dose panel in the submitted version is the same as the Medium-dose panel in the published version, but vertically stretched.
  3. The High-dose panel in Figure 5(a) in the submitted version is the same as the Low-dose panel in Figure 5(b) in the published version.

The authors do not agree with retraction and provided what they said were the correct files for Figures 2(b), 5(a), and 5(b).

## Supplementary Materials

Supplementary Materials The correct files for Figures 2(b), 5(a), and 5(b).
