# Supplementary figures and images for: Retracted: Topical Treatment with Xiaozheng Zhitong Paste (XZP) Alleviates Bone Destruction and Bone Cancer Pain in a Rat Model of Prostate Cancer-Induced Bone Pain by Modulating the RANKL/RANK/OPG Signaling
Source: Evid Based Complement Alternat Med. 2020 Dec 18;2020:1938781. doi: 10.1155/2020/1938781 (PMC7769638; doi:10.1155/2020/1938781)

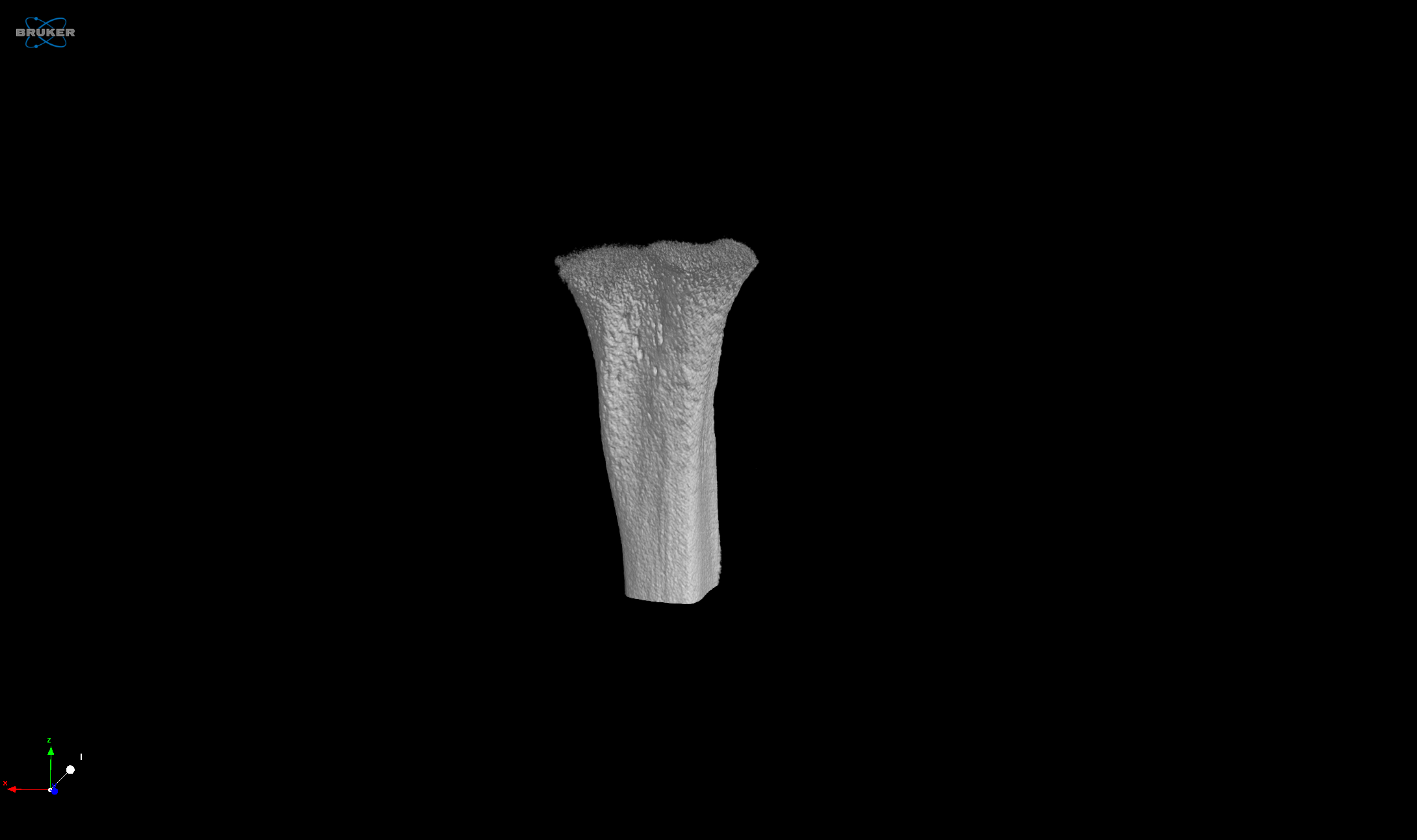

Supplement: Supplementary Materials — The correct files for Figures 2(b), 5(a), and 5(b). [file 1938781.f1.zip › 1938781.f1/Fig 2b/Control.bmp]

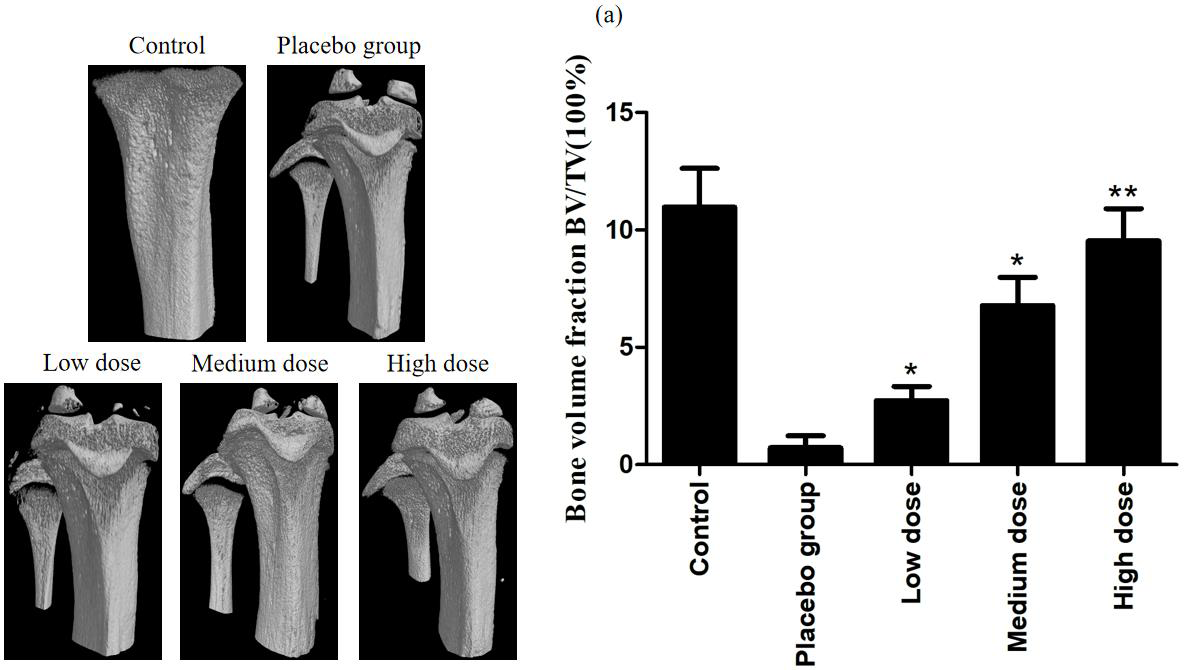

Supplement: Supplementary Materials — The correct files for Figures 2(b), 5(a), and 5(b). [file 1938781.f1.zip › 1938781.f1/Fig 2b/fig 2b.tif]

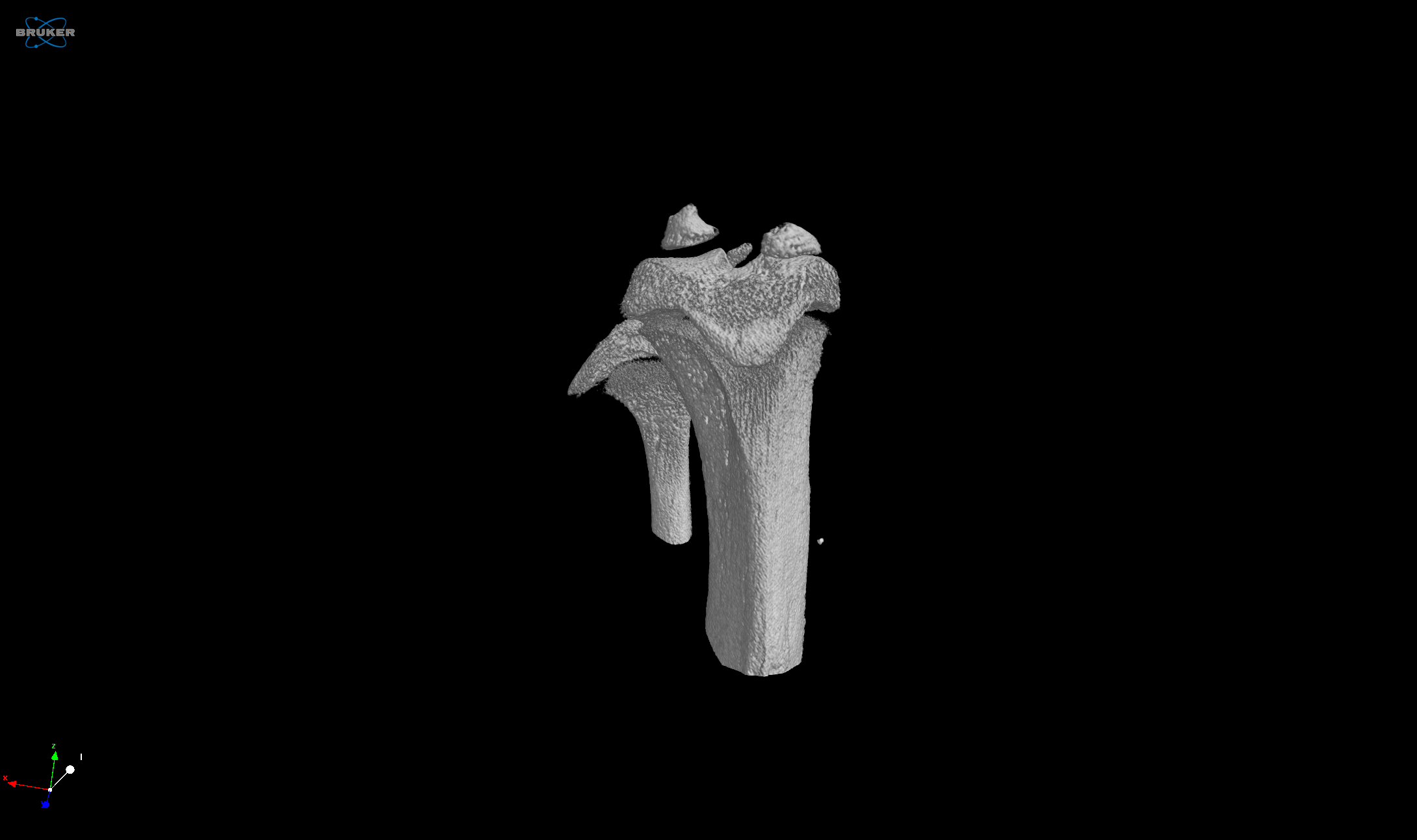

Supplement: Supplementary Materials — The correct files for Figures 2(b), 5(a), and 5(b). [file 1938781.f1.zip › 1938781.f1/Fig 2b/High dose.bmp]

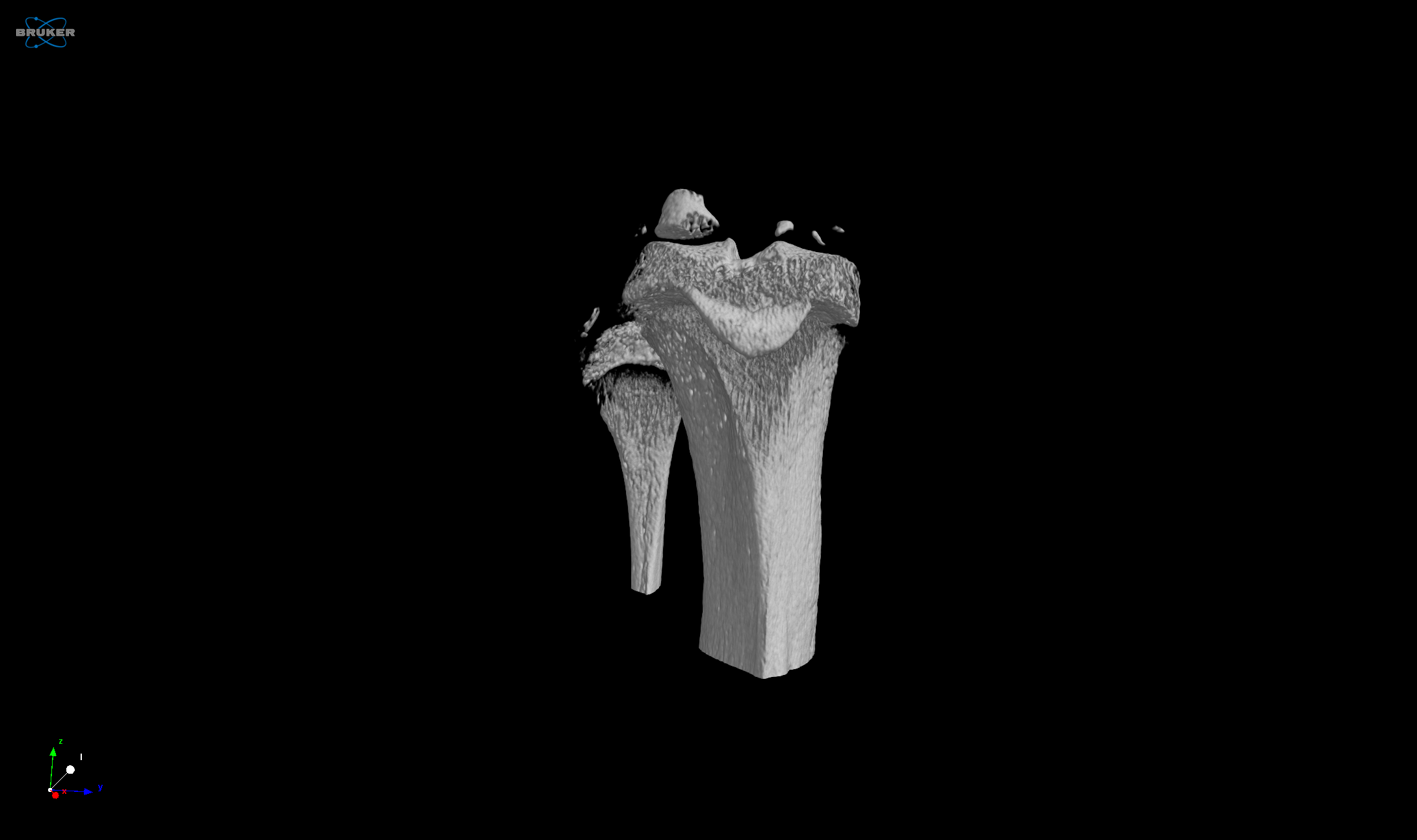

Supplement: Supplementary Materials — The correct files for Figures 2(b), 5(a), and 5(b). [file 1938781.f1.zip › 1938781.f1/Fig 2b/Low dose.bmp]

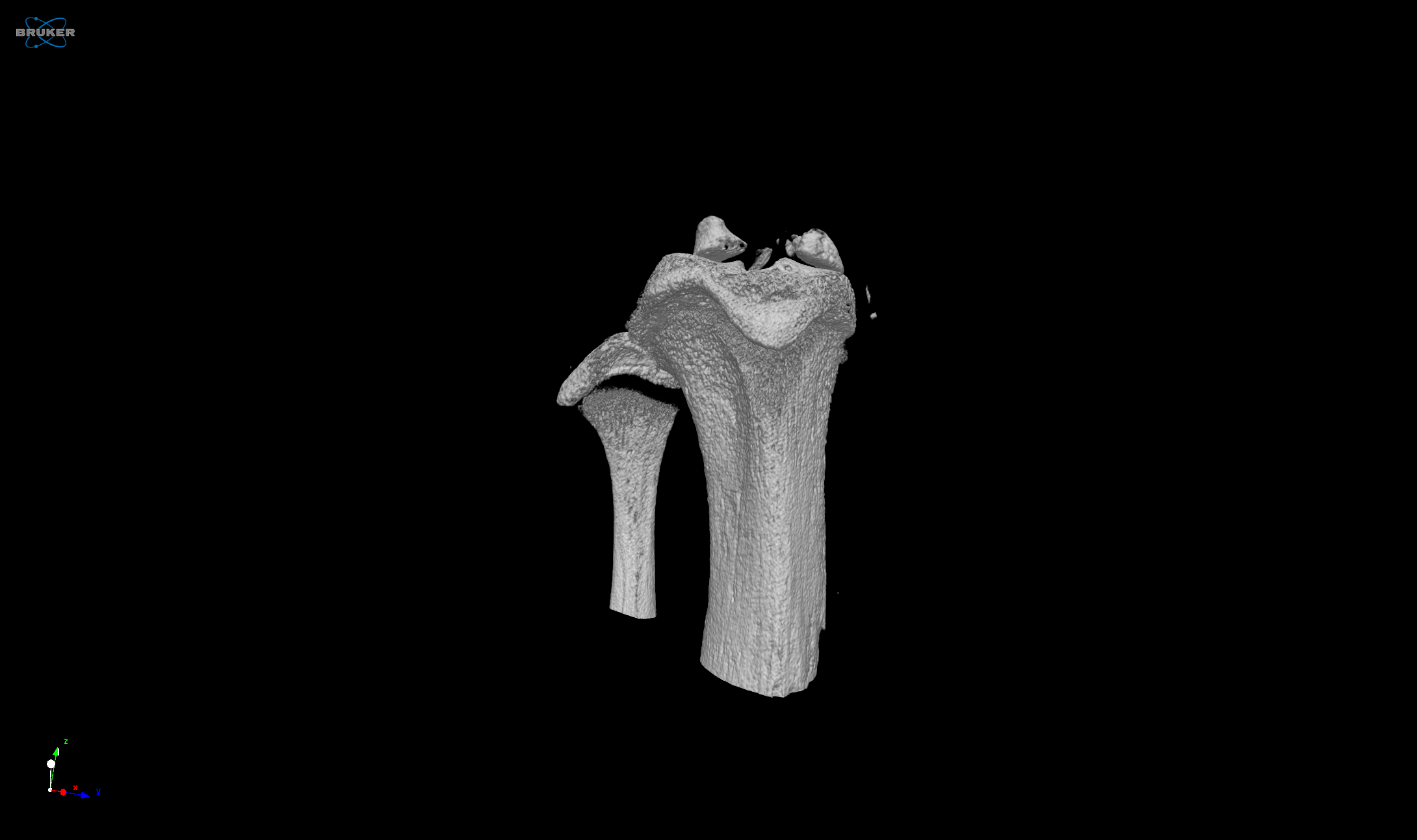

Supplement: Supplementary Materials — The correct files for Figures 2(b), 5(a), and 5(b). [file 1938781.f1.zip › 1938781.f1/Fig 2b/Medium dose.bmp]

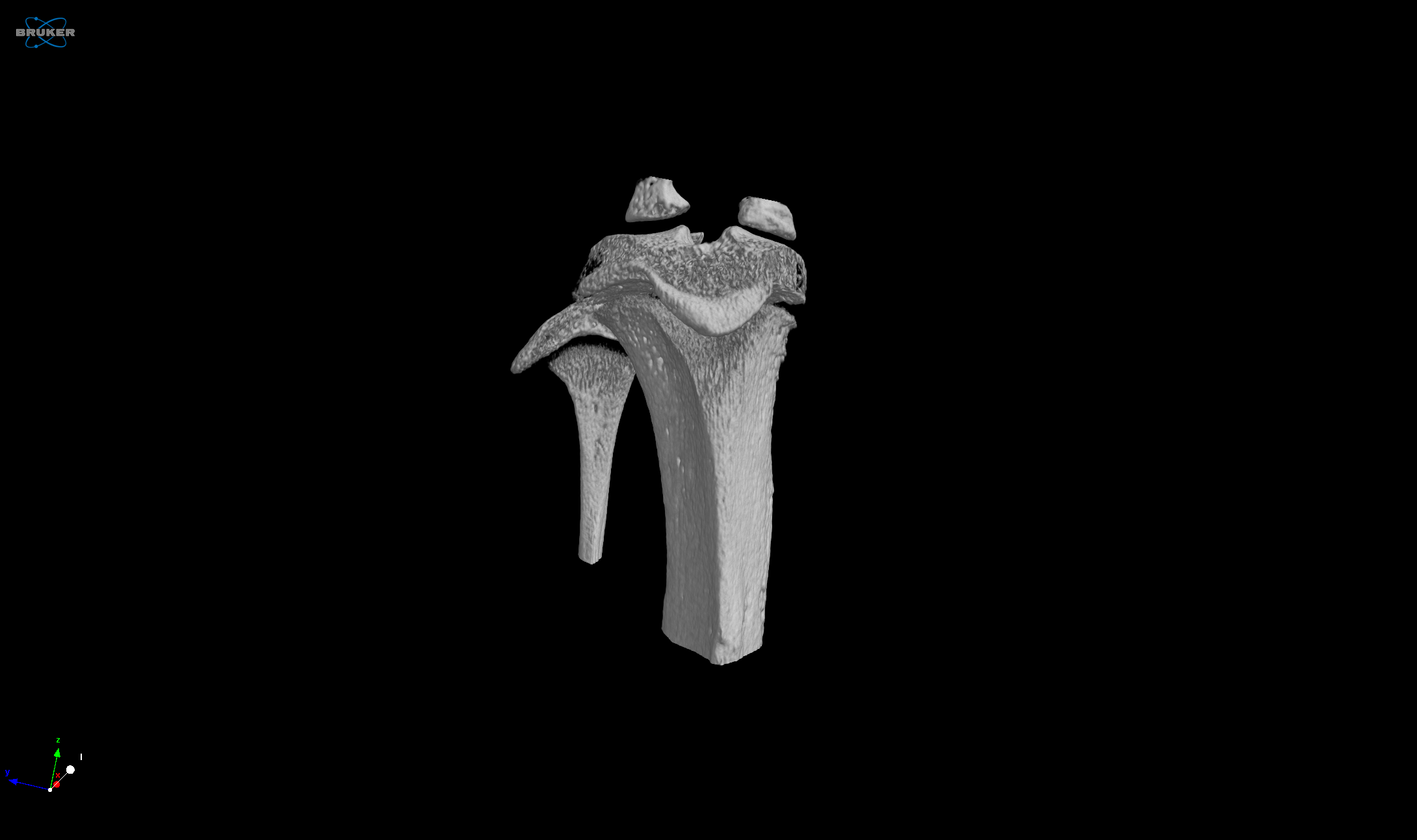

Supplement: Supplementary Materials — The correct files for Figures 2(b), 5(a), and 5(b). [file 1938781.f1.zip › 1938781.f1/Fig 2b/Placebo group.bmp]

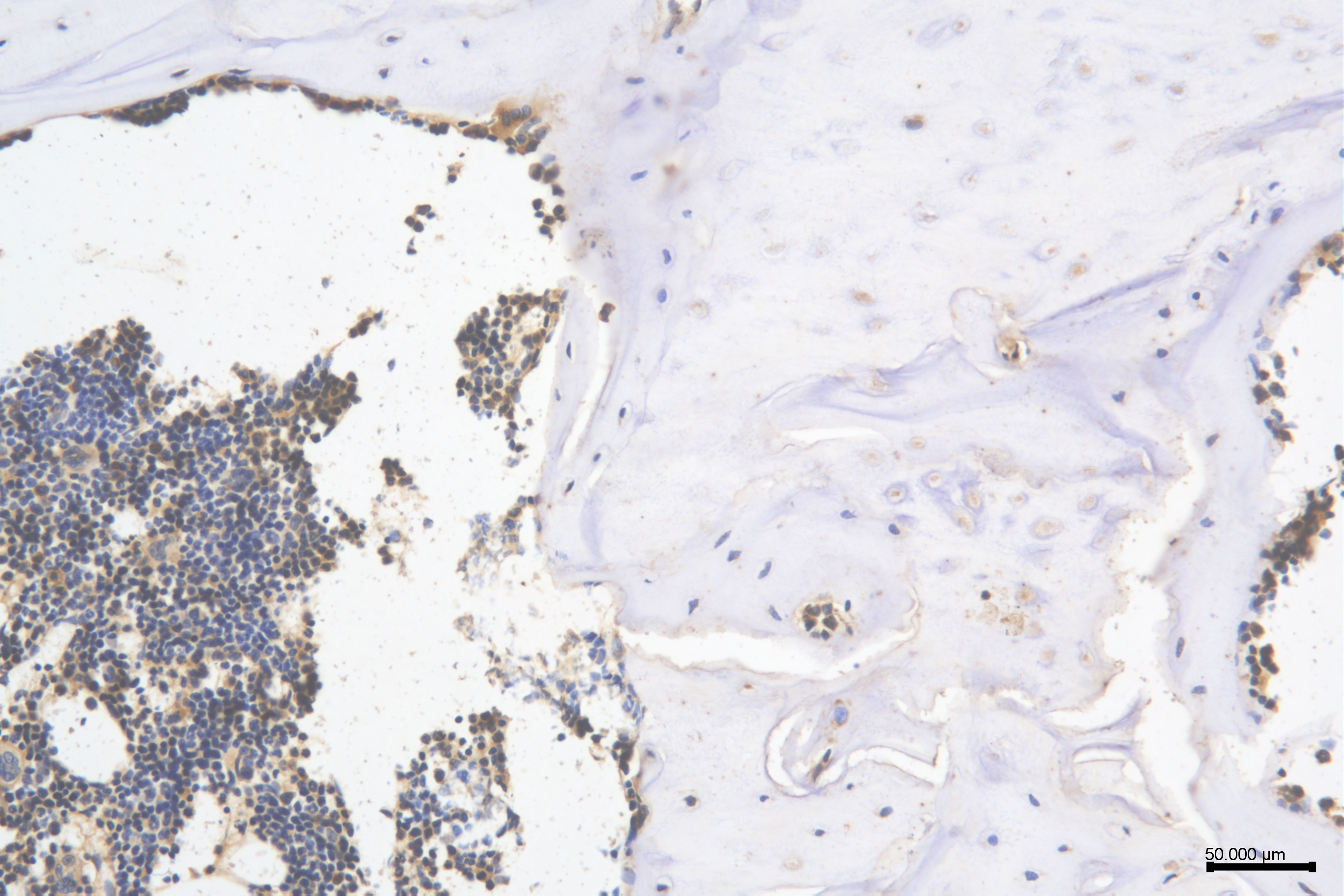

Supplement: Supplementary Materials — The correct files for Figures 2(b), 5(a), and 5(b). [file 1938781.f1.zip › 1938781.f1/Fig 5 a/Control.jpg]

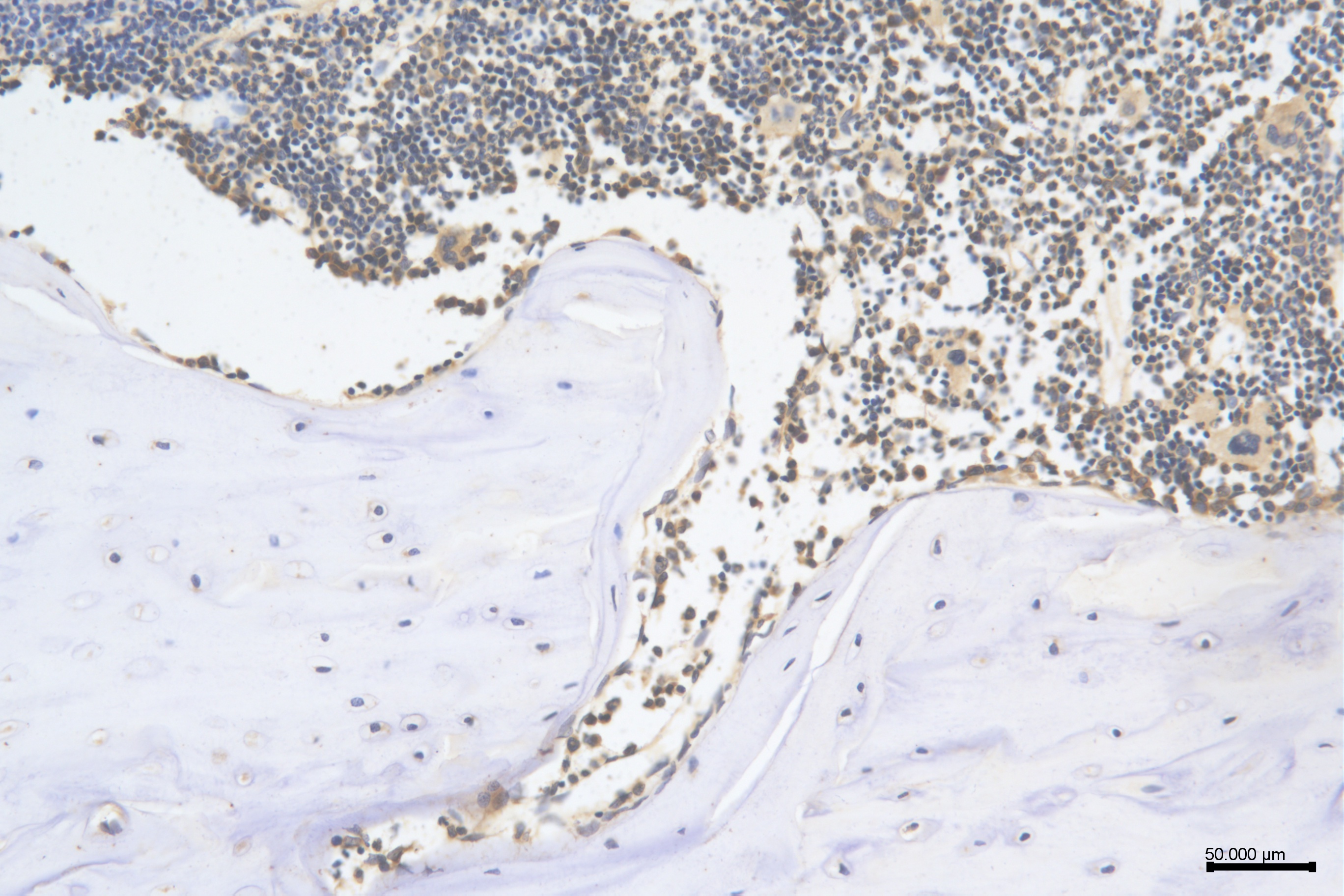

Supplement: Supplementary Materials — The correct files for Figures 2(b), 5(a), and 5(b). [file 1938781.f1.zip › 1938781.f1/Fig 5 a/High dose.jpg]

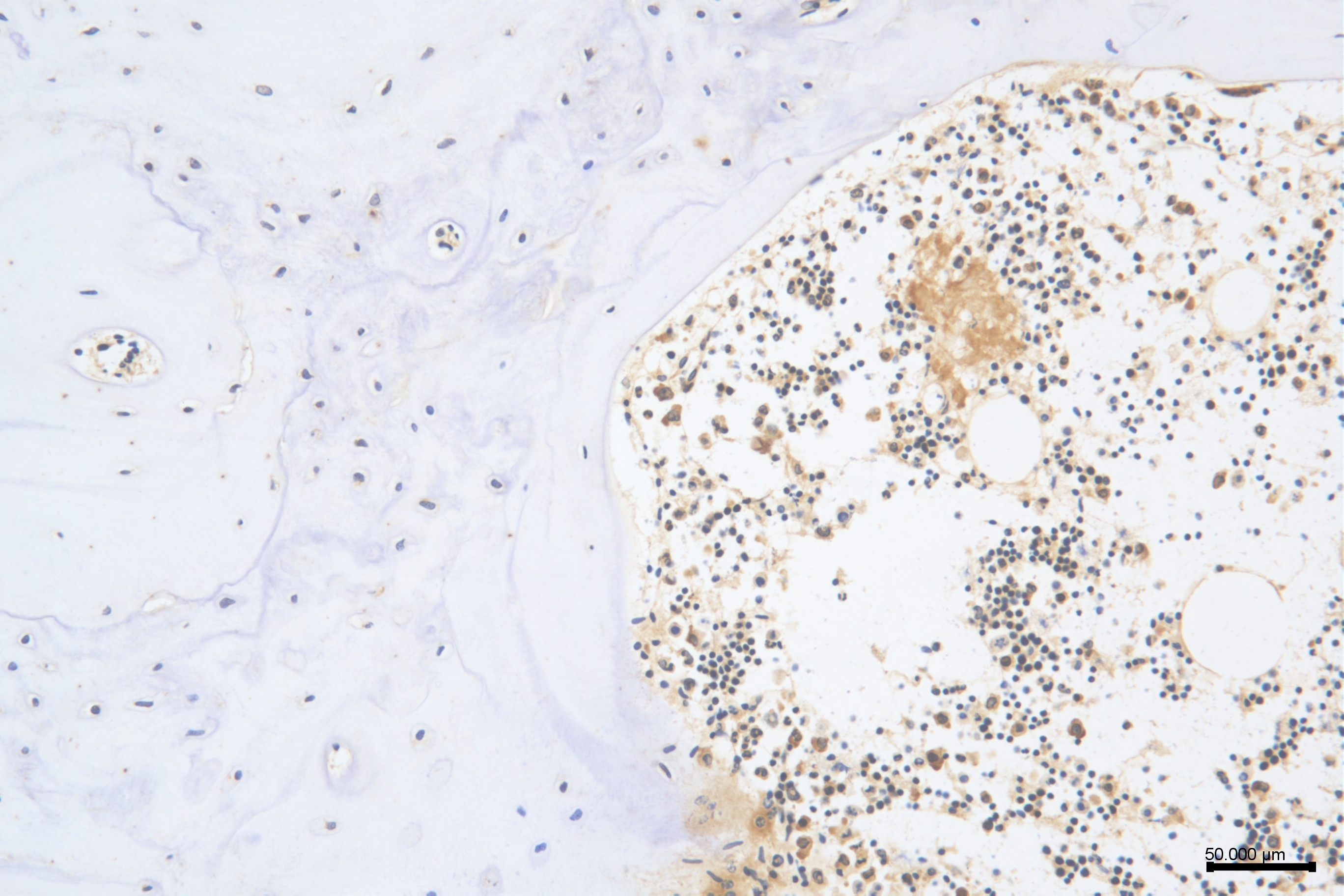

Supplement: Supplementary Materials — The correct files for Figures 2(b), 5(a), and 5(b). [file 1938781.f1.zip › 1938781.f1/Fig 5 a/Low dose.jpg]

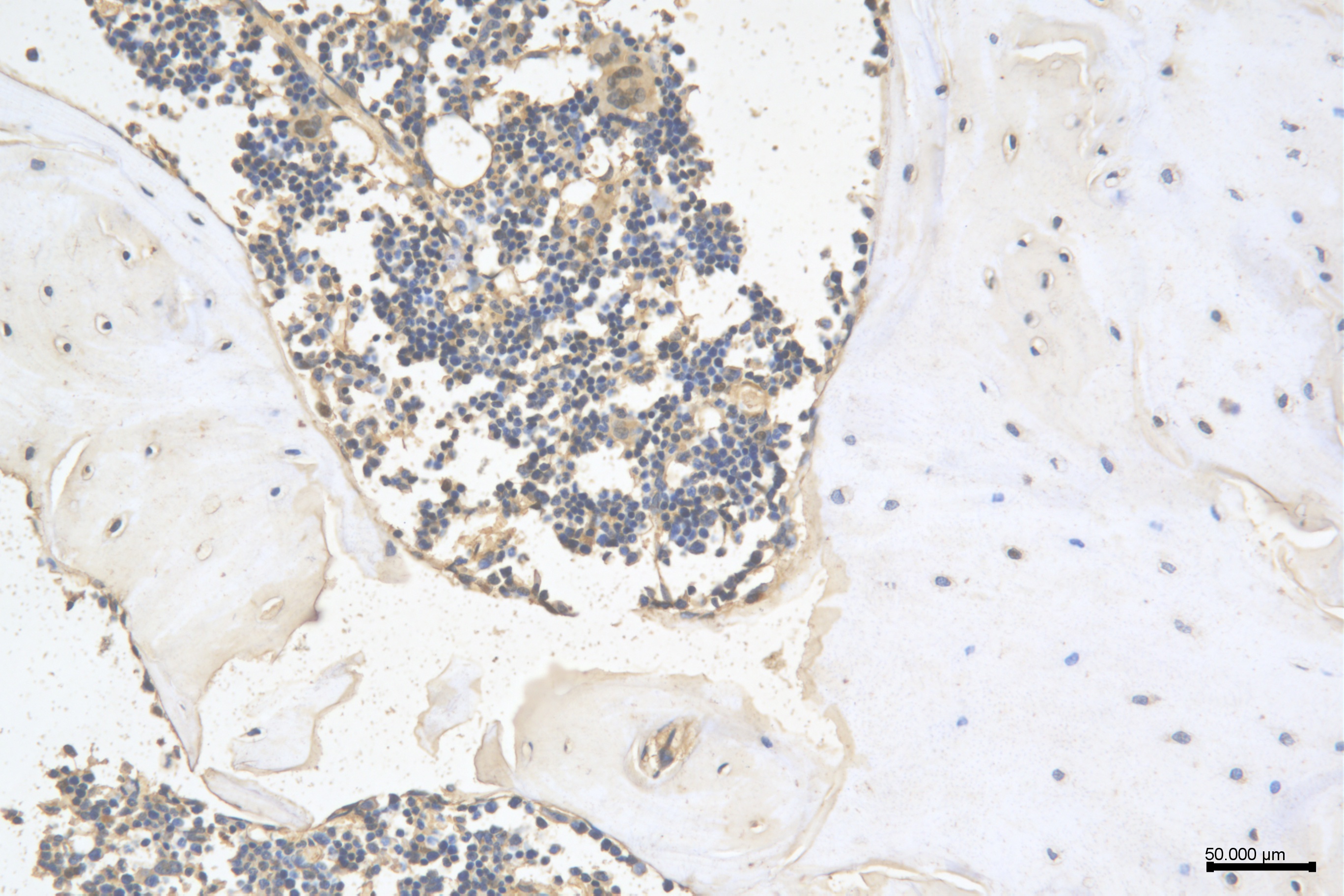

Supplement: Supplementary Materials — The correct files for Figures 2(b), 5(a), and 5(b). [file 1938781.f1.zip › 1938781.f1/Fig 5 a/Medium dose.jpg]

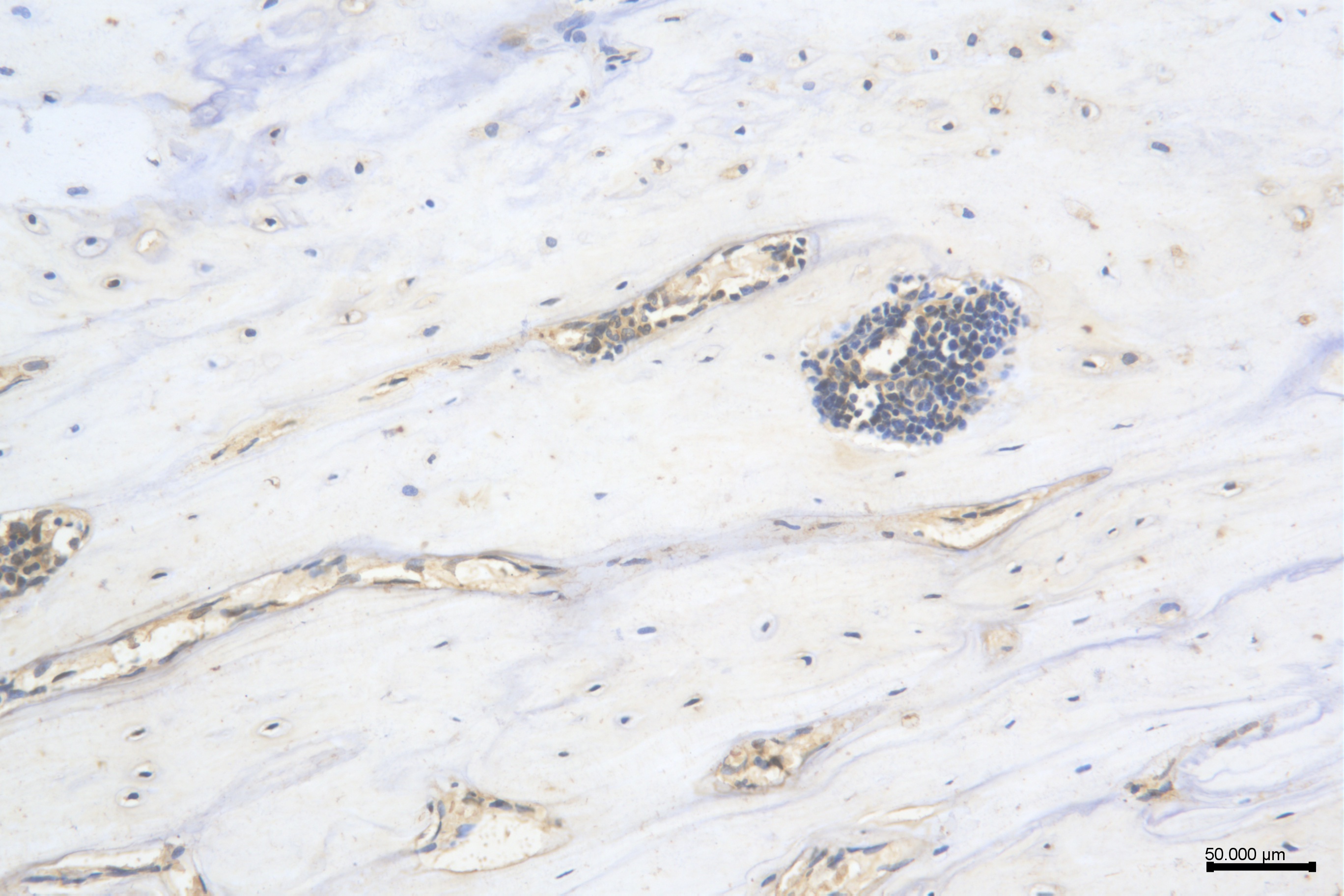

Supplement: Supplementary Materials — The correct files for Figures 2(b), 5(a), and 5(b). [file 1938781.f1.zip › 1938781.f1/Fig 5 a/Placebo.jpg]

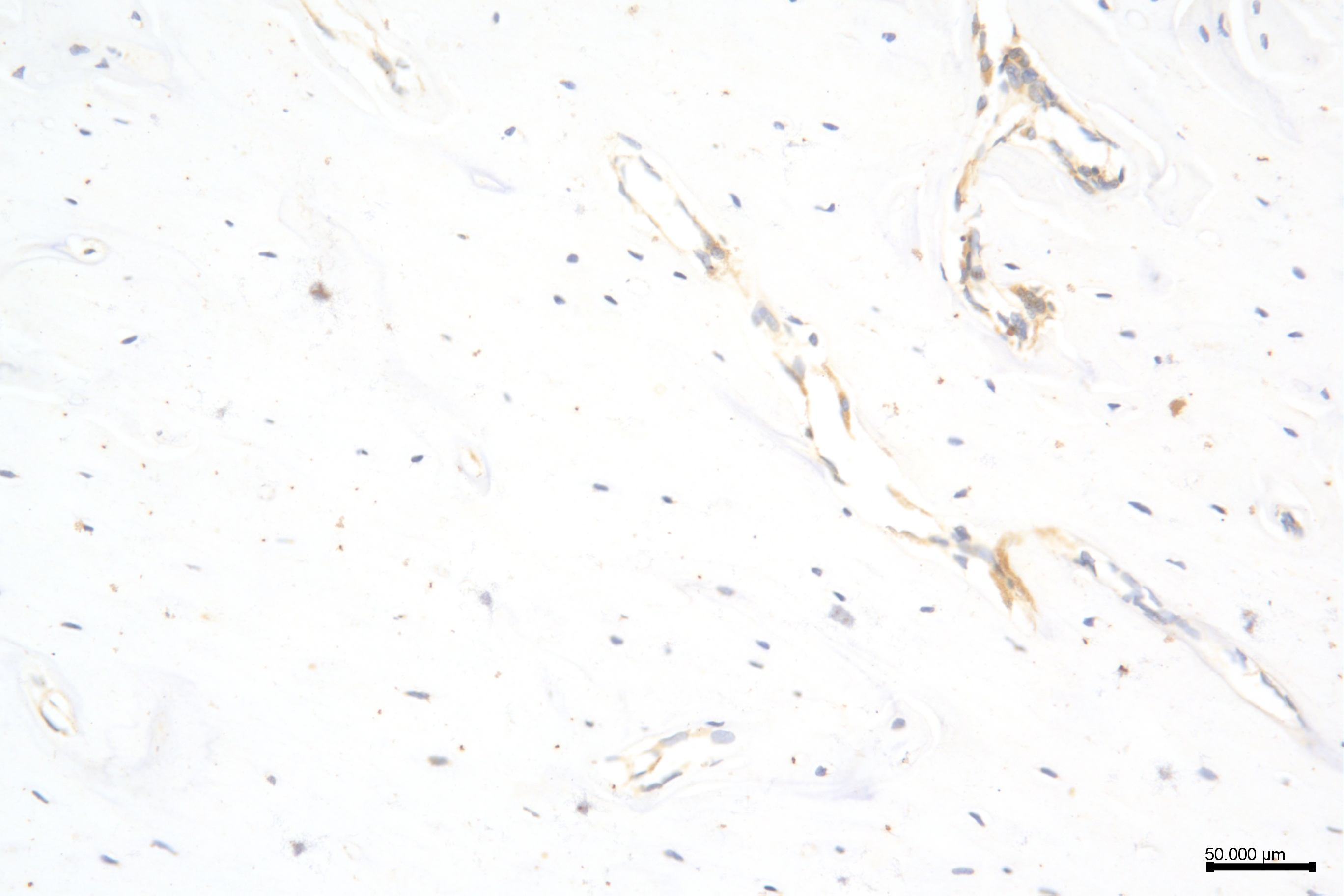

Supplement: Supplementary Materials — The correct files for Figures 2(b), 5(a), and 5(b). [file 1938781.f1.zip › 1938781.f1/Fig 5b/Control.jpg]

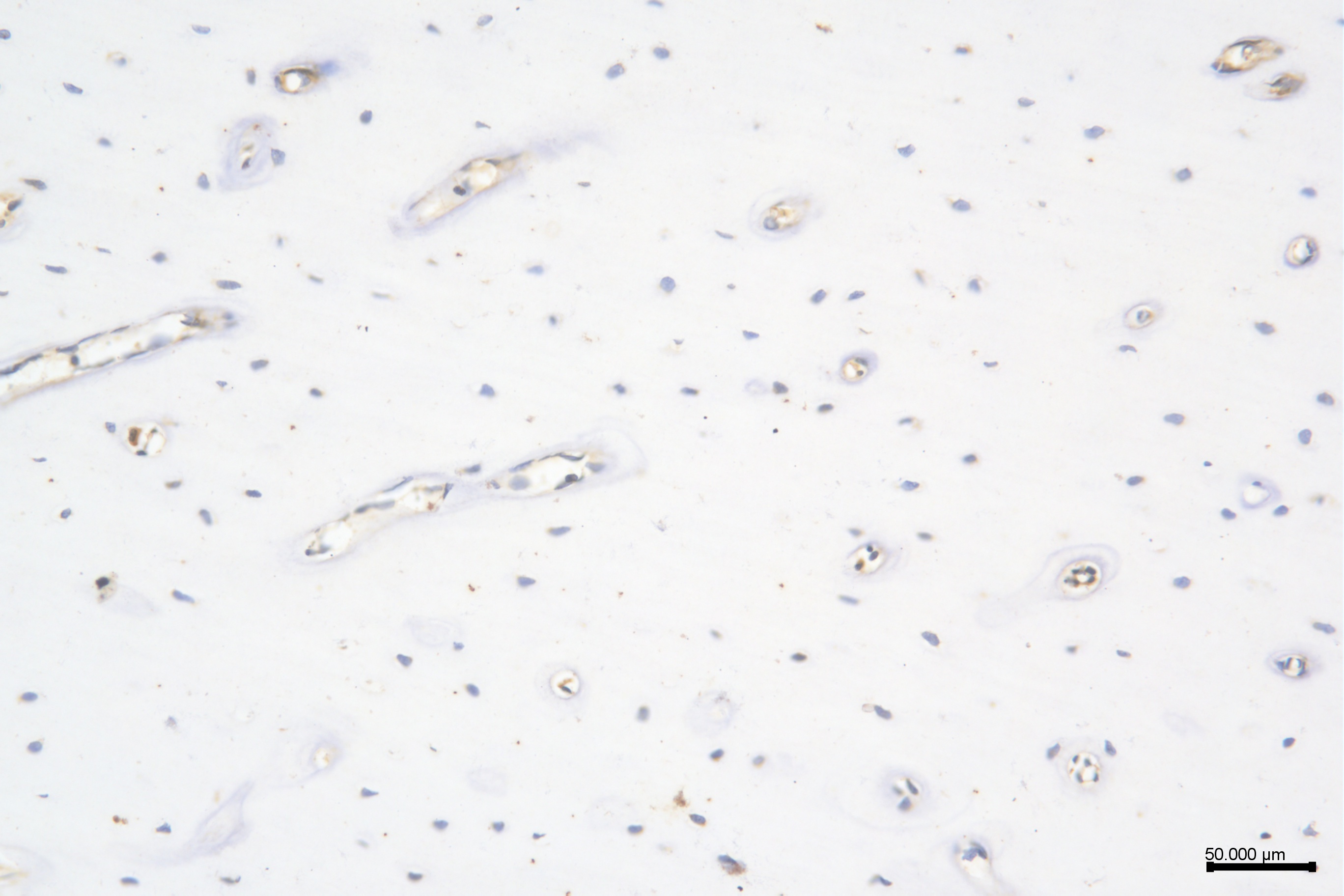

Supplement: Supplementary Materials — The correct files for Figures 2(b), 5(a), and 5(b). [file 1938781.f1.zip › 1938781.f1/Fig 5b/High dose.jpg]

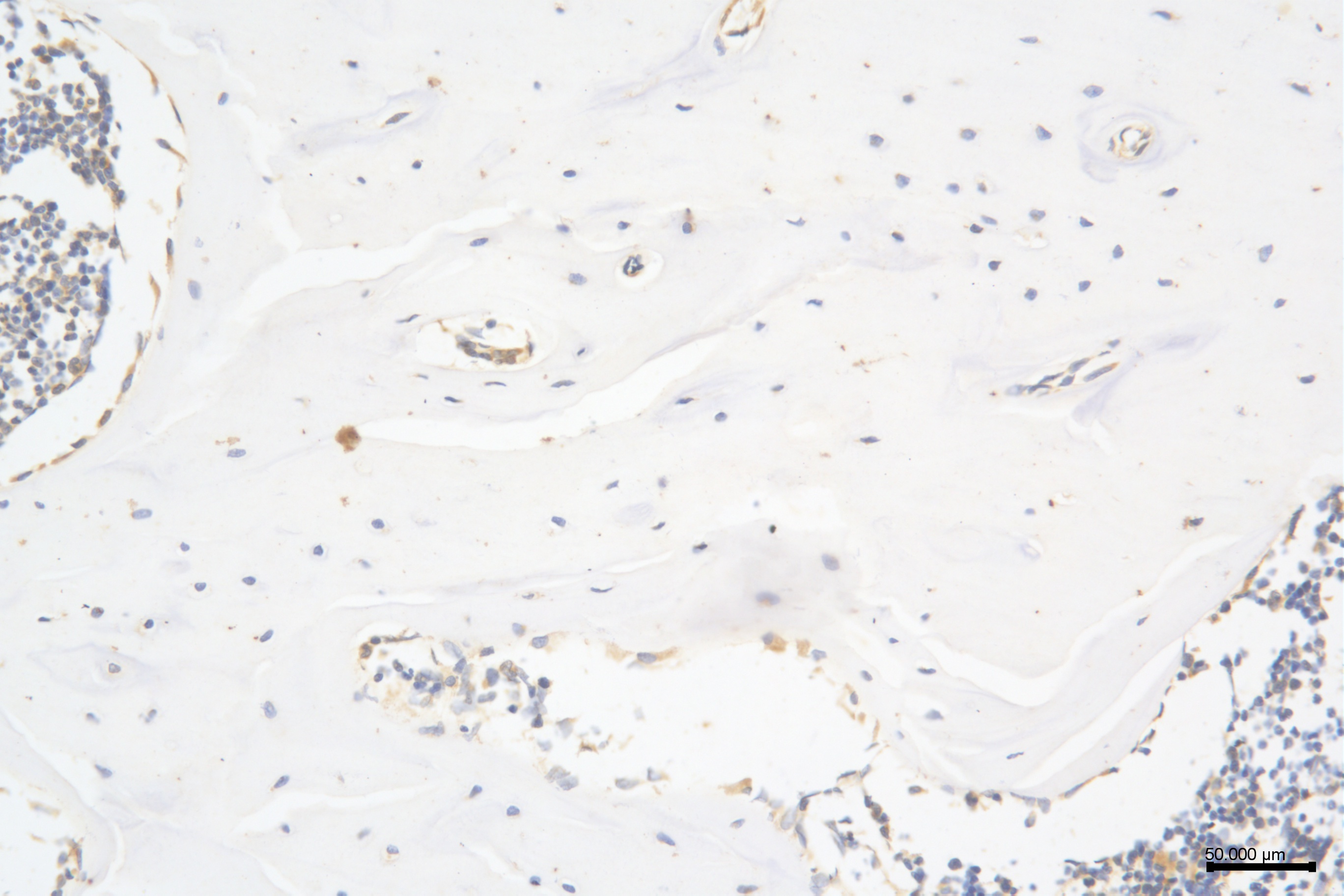

Supplement: Supplementary Materials — The correct files for Figures 2(b), 5(a), and 5(b). [file 1938781.f1.zip › 1938781.f1/Fig 5b/Low dose.jpg]

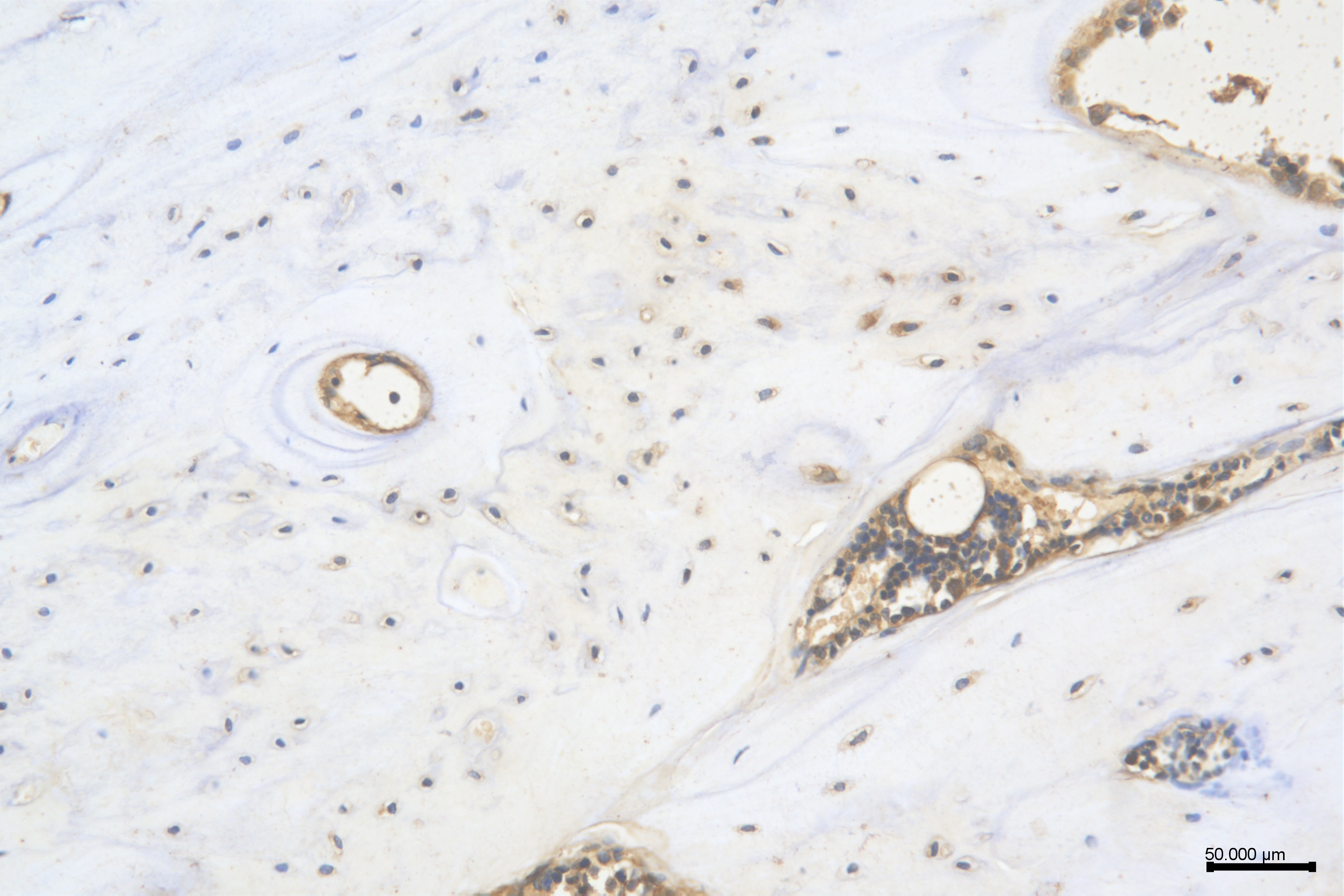

Supplement: Supplementary Materials — The correct files for Figures 2(b), 5(a), and 5(b). [file 1938781.f1.zip › 1938781.f1/Fig 5b/Medium dose.jpg]

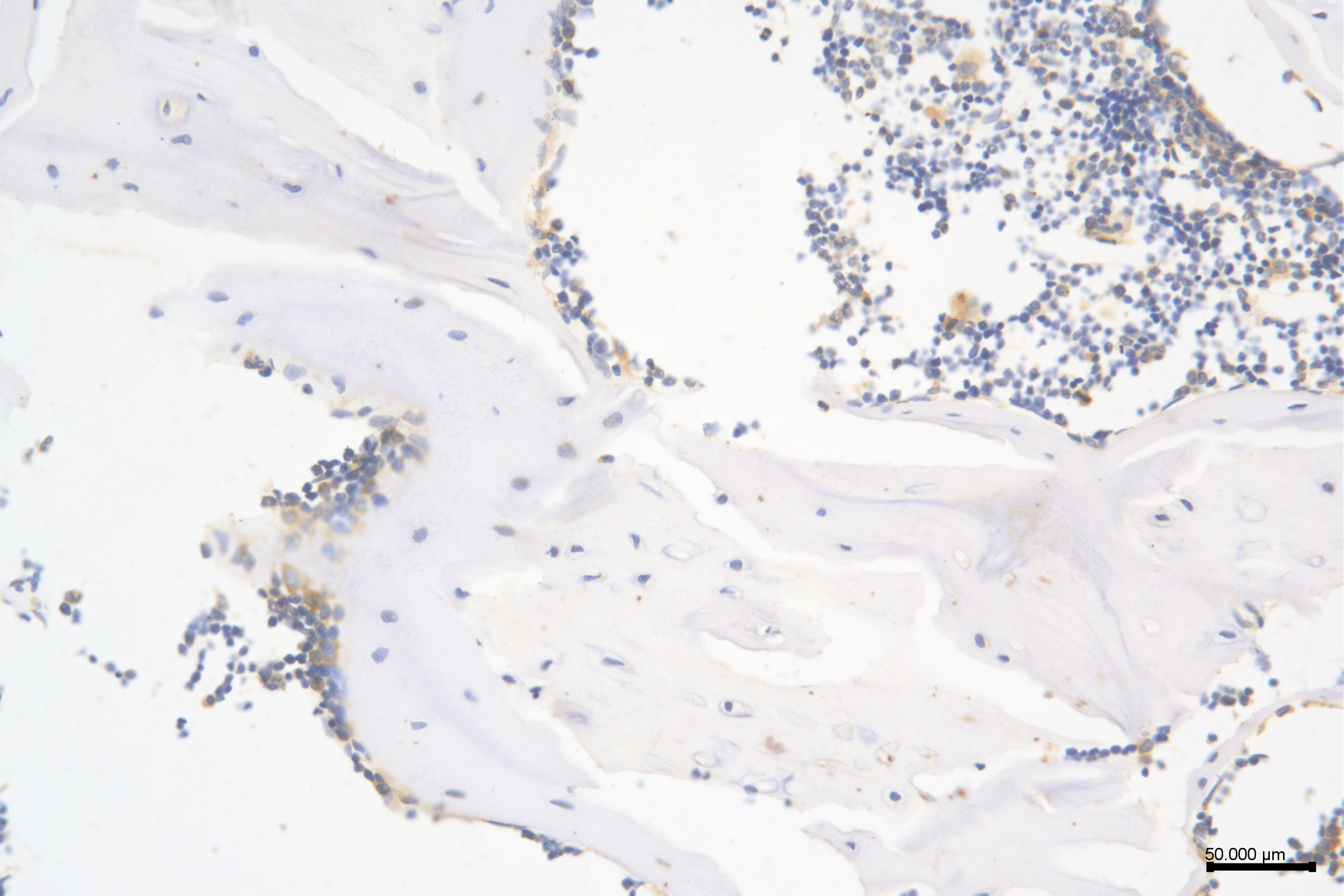

Supplement: Supplementary Materials — The correct files for Figures 2(b), 5(a), and 5(b). [file 1938781.f1.zip › 1938781.f1/Fig 5b/Placebo.jpg]
